# Supplementary material for: A simulation based difficult conversations intervention for neonatal intensive care unit nurse practitioners: A randomized controlled trial
Source: PLoS One. 2020 Mar 9;15(3):e0229895. doi: 10.1371/journal.pone.0229895 (PMC7062250; doi:10.1371/journal.pone.0229895)
Supplement: Attachment — Submitted filename: Reviwers comments.docx [file pone.0229895.s001.docx]

**Tiltle:** A simulation based difficult conversations intervention for NICU nurse practitioners: A randomized controlled trial

**Authors:** Roberta Bowen, Kate M Lally, Francine R Pingitore, Richard Tucker & Elisabeth C McGowan

**Overall comments**

Generally, the manuscrpit is well written with a good protocol. I will strongly recommend authors to look at the following comments and incorporate them.

Describe abbreviation fully when you are using them for the first time.

**Introduction**

The introduction part doesn’t show gap. You need to show the gap between existing situation and what the situation should be.

Also, show the severity of the problem on the parents and health care services.

**Materials and Methods**

Clearly state your measurements.

Describe also reliablity and validity of your measurements.

**Conclusion**

Describe the limitations of your study under this part.
